# System for Rapid, Precise Modulation of Intraocular Pressure, toward Minimally-Invasive *In Vivo* Measurement of Intracranial Pressure

**DOI:** 10.1371/journal.pone.0147020

**Published:** 2016-01-15

**Authors:** Max A. Stockslager, Brian C. Samuels, R. Rand Allingham, Zoe A. Klesmith, Stephen A. Schwaner, Craig R. Forest, C. Ross Ethier

**Affiliations:** 1 G. W. Woodruff School of Mechanical Engineering, Georgia Institute of Technology, Atlanta, GA, United States of America; 2 Department of Ophthalmology, University of Alabama at Birmingham School of Medicine, Birmingham, Alabama, United States of America; 3 Department of Ophthalmology, Duke University School of Medicine, Durham, NC, United States of America; 4 Coulter Department of Biomedical Engineering, Georgia Institute of Technology and Emory University, Atlanta, GA, United States of America; Casey Eye Institute, UNITED STATES

## Abstract

Pathologic changes in intracranial pressure (ICP) are commonly observed in a variety of medical conditions, including traumatic brain injury, stroke, brain tumors, and glaucoma. However, current ICP measurement techniques are invasive, requiring a lumbar puncture or surgical insertion of a cannula into the cerebrospinal fluid (CSF)-filled ventricles of the brain. A potential alternative approach to ICP measurement leverages the unique anatomy of the central retinal vein, which is exposed to both intraocular pressure (IOP) and ICP as it travels inside the eye and through the optic nerve; manipulating IOP while observing changes in the natural pulsations of the central retinal vein could potentially provide an accurate, indirect measure of ICP. As a step toward implementing this technique, we describe the design, fabrication, and characterization of a system that is capable of manipulating IOP *in vivo* with <0.1 mmHg resolution and settling times less than 2 seconds. *In vitro* tests were carried out to characterize system performance. Then, as a proof of concept, we used the system to manipulate IOP in tree shrews (*Tupaia belangeri*) while video of the retinal vessels was recorded and the caliber of a selected vein was quantified. Modulating IOP using our system elicited a rapid change in the appearance of the retinal vein of interest: IOP was lowered from 10 to 3 mmHg, and retinal vein caliber sharply increased as IOP decreased from 7 to 5 mmHg. Another important feature of this technology is its capability to measure ocular compliance and outflow facility *in vivo*, as demonstrated in tree shrews. Collectively, these proof-of-concept demonstrations support the utility of this system to manipulate IOP for a variety of useful applications in ocular biomechanics, and provide a framework for further study of the mechanisms of retinal venous pulsation.

## Introduction

The cerebrospinal fluid (CSF) serves as a mechanical cushion and chemical buffer for the tissues of the nervous system. The continual production and drainage of CSF generates a pressure known as the intracranial pressure (ICP). Alterations in ICP are a well-known feature of a variety of medical conditions including idiopathic intracranial hypertension [1], traumatic brain injury [2], hemorrhagic stroke [3], and brain tumors [4]. More recently, alteration of the translaminar pressure difference, the difference between intraocular pressure (IOP) and ICP, has emerged as an important risk factor for open-angle glaucomas [5]; in particular, ICP is low in patients with normal-tension glaucoma, an important and frequently unrecognized subset of this blinding disease [6]. ICP alterations have also been hypothesized to be involved in the visual impairment experienced by astronauts after prolonged exposure to microgravity [7], and a better understanding of this phenomenon is a major focus of investigation for space programs.

Several techniques exist for measuring ICP. In extreme cases, a craniotomy is performed and the CSF-filled ventricles of the brain are accessed directly with a cannula. Alternatively, ICP can be measured by performing a lumbar puncture (or “spinal tap”), in which a needle is inserted between the lumbar vertebrae to access the subarachnoid space at the base of the spinal cord. Lumbar puncture has been associated with severe headache in up to 30% of patients [8], cerebrospinal fluid leaks [9], and herniation of the brain stem into the foramen magnum [10]. Despite these clinically significant risks, over 500,000 lumbar puncture procedures are performed annually in the U.S. to measure ICP, to sample CSF composition, or both. Thus, it is generally agreed that there is a need for a less invasive method of ICP measurement.

A number of technologies are under development to meet this need, such as detecting movement of the tympanic membrane [11]. However, thus far, these noninvasive technologies have yet to reach the clinic, due in part to concerns about their accuracy. The accuracy and resolution of ICP measurements are of great importance in clinical settings; for example, the mean ICP difference between normal-tension glaucoma patients and controls may be as little as 2.7 mmHg [6]. However, achieving this level of accuracy and resolution is particularly challenging for noninvasive methods, since all such approaches rely on population-averaged assumptions about the mechanical properties of the tissues being interrogated, which can vary significantly from patient to patient [12].

The central retinal vein has been suggested as an attractive alternative target for interrogating ICP because it possesses a unique combination of features: (1) it is exposed to both IOP and ICP as it passes inside the eye and through the optic nerve, respectively; (2) due to the vein’s compliance, its shape is determined by the balance of these pressures; and (3) the vein can be viewed within the eye noninvasively, using a slit lamp or indirect ophthalmoscopy [13].

The time-varying balance between IOP and ICP throughout the cardiac cycle causes the central retinal vein to visibly pulsate when IOP and ICP are normal. When ICP is elevated or IOP is lowered, however, no pulsations are observed [13]. The details of the physical mechanism responsible for retinal venous pulsation are not fully understood. Levine proposed a model in which the pulsations result from periodic variations in the translaminar pressure difference [14]. This model is based on the fact that IOP experiences larger fluctuations than ICP during the cardiac cycle, with amplitudes of 1.5 mmHg and 0.5 mmHg respectively [15]. The resulting oscillations in the translaminar pressure difference result in accumulation (during diastole) and drainage (during systole) of blood from the intraocular portion of the central retinal vein, causing the vein to visibly dilate and collapse. According to this model, pulsations are lost at high ICP or low IOP because raising ICP or lowering IOP also changes the amplitude of translaminar pressure difference oscillations. The phenomenon of retinal venous pulsation has also been studied *in vivo*: changes in retinal venous pulsation that occur as a result of manipulating ICP have been investigated in some detail in a dog model [16].

Observing the presence or absence of retinal venous pulsation can provide an estimate of ICP. This approach is already used clinically in a qualitative manner, where lack of spontaneous retinal venous pulsation is an indicator of elevated ICP [13]. To improve upon this approach, the IOP dependence of retinal venous pulsation could be exploited to provide a more quantitative measurement of ICP: lower IOP until the central retinal vein stops pulsating, then use this IOP value to deduce ICP.

Golzan et al. [17] investigated this ICP measurement technique in human subjects, estimating ICP by artificially lowering IOP while monitoring retinal venous pulsation amplitude. The authors applied topical apraclonidine to gradually lower IOP over 45 minutes, and measured IOP using a Goldmann tonometer in 15 minute increments. The resulting noninvasive ICP estimates differed from invasive ICP measurements by -3.07 mmHg to 4.93 mmHg. Although superior to many other noninvasive ICP measurement approaches, these measurements are time consuming to perform and lack sufficient accuracy for clinical use in glaucoma management [6]. The relative lack of accuracy may be attributed in part to the technique used for IOP assessment. While Goldmann tonometry is the “gold standard” for clinical measurement of IOP [12], limitations on its accuracy [18] and repeatability [19] have been well documented. For example, Wang et al. [19] showed that repeated IOP measurements by the same operator on the same patients differ by as much as 4.34 mmHg. This IOP measurement error is comparable to the ICP measurement errors observed by Golzan et al. [17], suggesting that with more reliable IOP measurements, the retinal venous system could provide significantly more accurate ICP estimates. Another limitation of the study was the method used to manipulate IOP; it is not possible to accurately predict the rate at which pharmacologic treatments such as apraclonidine lower IOP for individual subjects. An improved method to accurately and repeatably measure and manipulate IOP would therefore be desirable.

To address weaknesses in current approaches to manipulating IOP *in vivo*, we have designed, fabricated, and tested a “minimally invasive” IOP modulation system, capable of modulating and directly measuring IOP with < 0.1 mmHg resolution with settling times on the order of 2 seconds. IOP control was accomplished by injecting and withdrawing small volumes of fluid from the eye, using a fine-bore needle that passes through the peripheral cornea and into the fluid-filled anterior chamber. To characterize system performance, *in vitro* testing was performed using an artificial eye and enucleated porcine eyes. Then, as a proof of concept, we used the system to manipulate IOP *in vivo* in tree shrews to elucidate visible changes in the retinal vasculature. As a secondary application for this system, we also used the tree shrew model to interrogate two other properties of the eye of importance in glaucoma: the ocular compliance [20] and outflow facility [21]. Preliminary testing has indicated that this system is superior in accuracy and dynamic response to current approaches for *in vivo* IOP manipulation.

## Methods

### System design

**Fig 1:**
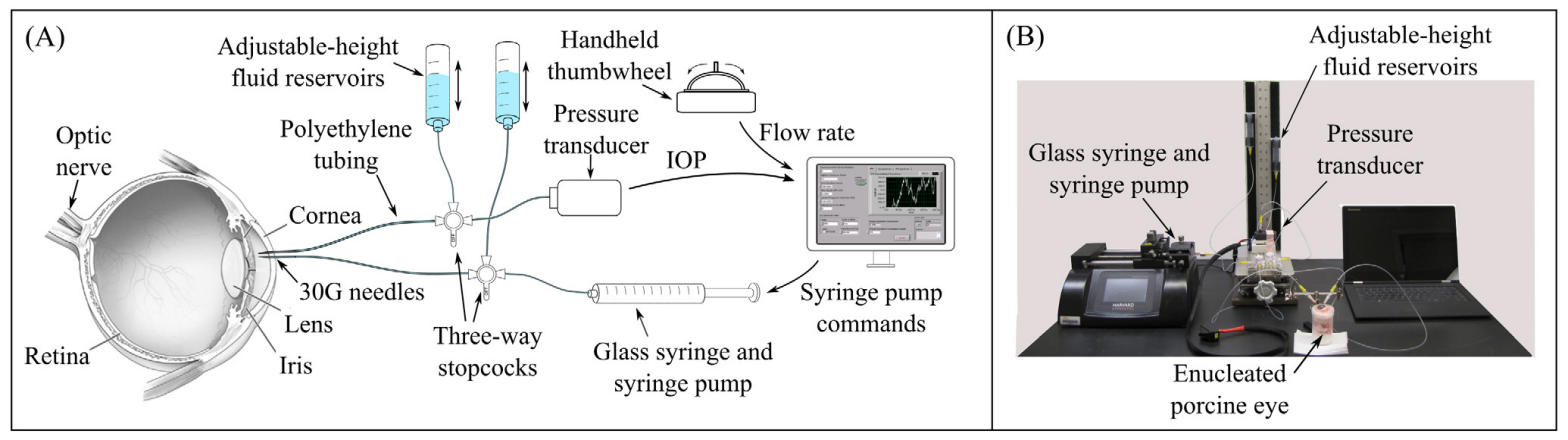
Schematic of IOP modulation system. (a) A syringe pump injected or withdrew fluid from the eye via a fine-bore needle inserted into the anterior chamber through the peripheral cornea, while a second needle connected to a pressure transducer recorded IOP. (b) Photo of system as assembled, with a cannulated enucleated porcine eye.

IOP was modulated by inserting a 30-gauge beveled needle (outer diameter 0.31 mm) into the anterior chamber near the lateral corneal limbus, and injecting or withdrawing Dulbecco’s phosphate buffered saline (PBS) with added 5.5 mM glucose using a 100 μL syringe (Hamilton) actuated by a syringe pump (Harvard Apparatus PHD Ultra; Fig 1). A second 30-gauge beveled needle was connected to a pressure transducer (Honeywell 142PC01G) and also inserted into the anterior chamber to record IOP in real time. Using this second needle as a static pressure tap was suitable for this prototype system, as it ensured that the pressure transducer always recorded the true IOP; if the pressure transducer were connected in parallel with the syringe pump using a single needle, the measured pressure would be the sum of the true IOP and the pressure drop across the tubing and needle. Fluid connections were made using semi-rigid polyethylene tubing (inner diameter 0.86 mm, BD PE-100). A pair of adjustable-height PBS-filled reservoirs allowed calibration of the pressure transducer, refilling the syringe, or manipulation of IOP, depending on the orientation of the three-way stopcocks.

A handheld, spring-loaded thumbwheel (Megatron TRY13) with proportional voltage output was used for manual control of the syringe pump flow rate, which enabled open-loop IOP control as described in “System characterization.” In this mode, syringe pump flow rate was proportional to the displacement of the thumbwheel from its neutral position in either the positive (inject) or negative (withdraw) directions.

The syringe pump was controlled using a computer interface developed in LabVIEW. Pressure transducer data was acquired at 10 Hz using a 12-bit DAQ board (NI USB-6008). Syringe pump commands were sent at 10 Hz using serial communication, via USB configured as a virtual COM port.

### Lumped parameter model

**Fig 2:**
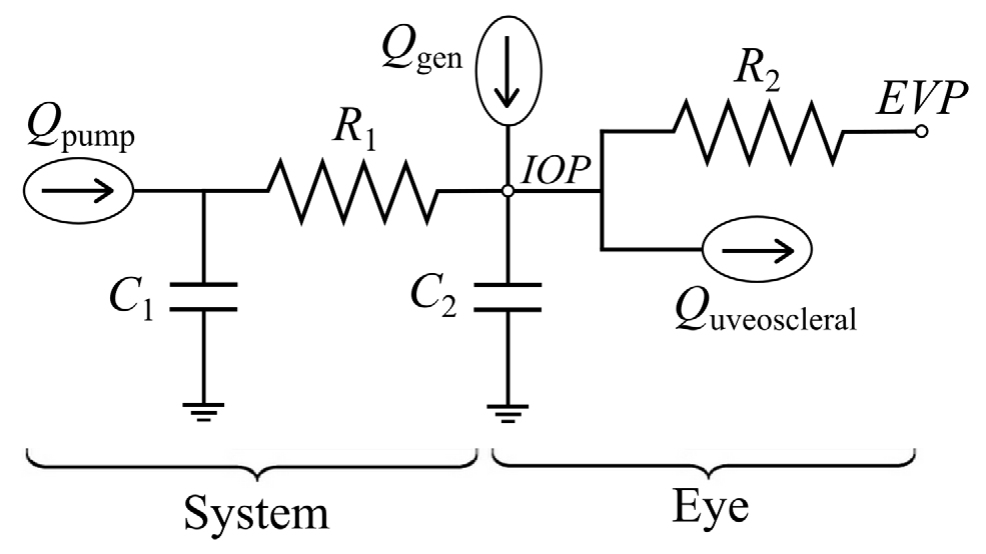
Lumped parameter model of system and ocular fluid dynamics.

In order to predict the IOP changes resulting from fluid injections or withdrawals, the overall fluid dynamic behavior of the system and the eye was approximately by a lumped parameter model (Fig 2). The syringe pump provided flow at volumetric flow rate *Q*_pump_. The system was represented by volumetric compliance *C*_1_ in parallel with flow resistance *R*_1_. The fluid dynamic properties of the eye were represented by the ocular compliance *C*_2_ and aqueous humor generation rate *Q*_gen_ along with two parallel outflow pathways. Conventional outflow was characterized by conventional outflow resistance *R*_2_ and the episcleral venous pressure (*EVP*); EVP is essentially the back-pressure against which fluid drains from the eye *in vivo*. Uveoscleral outflow, which is commonly approximated as independent of IOP [22–24], was represented by constant outflow rate *Q*_uveoscleral_. The pressure transducer measured IOP. The model was reduced to a set of ordinary differential and algebraic equations using techniques equivalent to electrical circuit analysis.

#### Ocular compliance measurement

Ocular compliance is defined as the increase in ocular volume per unit increase in IOP [25]. It serves as a simple proxy for mechanical properties of the eye which are more difficult to measure, such as the stiffness of the sclera, which is thought to be important in glaucoma [26].

Ocular compliance, represented by *C*_2_ in the lumped parameter model (Fig 2), was measured by injecting a fluid bolus of volume Δ*V* and measuring the resulting IOP increase, Δ*IOP*. Assuming that a negligible volume of fluid flows out of the eye during this rapid injection, the ocular compliance was given by

$$C_{2}=\frac{\Delta V}{\Delta IOP}-C_{1}$$ (1)

where *C*_1_, the system compliance, was independently characterized (see “System volumetric compliance measurement”).

#### Outflow facility measurement

Outflow facility is defined as the change in aqueous humor volumetric outflow rate per unit increase in IOP. Low outflow facility is associated with glaucoma, and some glaucoma treatment approaches aim to increase outflow facility [21]. Here we consider the conventional outflow facility, which is the primary variable of interest in explaining elevated IOP [27]. Its reciprocal, the conventional outflow resistance, appears as *R*_2_ in the model (Fig 2). Using the model, the IOP decay following a fluid injection was given by

$$IOP=EVP+(\Delta IOP)e^{-t/R_{2}(C_{1}+C_{2})},$$ (2)

where Δ*IOP* is the magnitude of the IOP increase following the fluid injection. To calculate the conventional outflow resistance *R*_2_, a multi-parameter least squares best fit was performed to fit the IOP vs. time trace to a function of the form

$$IOP=K_{1}+K_{2}e^{-t/\tau },$$ (3)

with *K*_1_, *K*_2_, and τ as free parameters. Comparing Eqs 2 and 3, the conventional outflow resistance was then given by

$$R_{2}=\frac{\tau }{C_{1}+C_{2}}$$ (4)

with the conventional outflow facility simply the reciprocal of *R*_2_.

### System characterization

The system was used to manipulate IOP using three different modes: bolus injection, closed-loop IOP control, and open-loop IOP control. Injecting or withdrawing fluid from the eye in each of these modes allowed different measurements to be made.

In bolus injection mode, a fixed volume of fluid was rapidly injected (typically over 1 second), causing a rapid increase in IOP followed by a gradual decay as fluid drained from the eye. Ocular compliance and outflow facility were deduced from the resulting IOP vs. time trace, using Eqs 1 and 4 respectively.

In closed-loop IOP control mode, the syringe pump flowrate was adjusted via proportional feedback control to bring IOP to a desired set point. Here we demonstrate closed-loop IOP control as a proof of concept in enucleated porcine eyes. We did not use this mode *in vivo* in tree shrews, since it would not preserve the natural IOP oscillations which are responsible for retinal venous pulsation.

In open-loop IOP control mode, the syringe pump flowrate was controlled manually by the user using the handheld spring-loaded thumbwheel (Fig 1), with flowrate proportional to the displacement of the thumbwheel from its neutral position. This mode allowed manipulation of mean IOP while preserving its natural oscillations *in vivo*. As a simpler alternative to using the syringe pump, open-loop IOP control was also accomplished by manually adjusting the height of a PBS-filled fluid column connected to the eye. However, a thumbwheel control or equivalent would be desired for eventual clinical use of the system.

#### Pressure transducer calibration

A five-point calibration was performed on the pressure transducer before each use by connecting the transducer to a PBS-filled fluid column of adjustable height. For each calibration point, the free surface of the fluid column was set to a known height relative to the pressure transducer, generating a known hydrostatic pressure, and the voltage output of the pressure transducer was recorded. A line was fit to the pressure vs. voltage calibration data, and the calibration was considered successful if *R*^2^ > 0.9999; otherwise the fluid connections were checked for air bubbles and the calibration was repeated.

After performing this calibration, the free surface of the fluid column was set to the height of the limbus of the eye and the pressure transducer output was recorded. This datum pressure was subtracted from all future IOP data to account for the hydrostatic pressure difference between the eye and the pressure transducer.

#### System volumetric compliance measurement

Accounting for the volumetric compliance of the system is necessary for calculation of the ocular compliance and outflow facility using Eqs 1 and 4. To measure this compliance, *C*_1_ in the lumped parameter model (Fig 2), the pump and pressure transducer lines were connected together to seal off the system. The system was then pressurized by injecting a volume, Δ*V*, causing a pressure increase of magnitude Δ*P*. The system compliance was calculated as the ratio *C*_1_ = Δ*V* / Δ*P*. Lower system compliance is desirable in this case, in order to increase the signal-to-noise ratio in ocular compliance measurements.

#### Artificial eye measurements

For benchtop testing of the system, an artificial eye was assembled by connecting a glass capillary (CM Scientific, length 90 mm, inner diameter 51 μm) in parallel with a length of compliant polyethylene tubing (Tygon, length 200 mm, inner diameter 1.65 mm) using a three-way Luer connector. The tubing was sealed at the free end using a stopcock. This arrangement simulated the parallel outflow resistance and compliance of an eye. The artificial eye was filled with PBS, and the syringe pump and pressure transducer lines were connected to the eye using a second three-way Luer connector. The free end of the capillary was submerged in PBS to negate the effects of surface tension on fluid outflow; this free surface was used as the datum for the pressure transducer.

To measure the compliance and outflow facility of the artificial eye, a 1 μL bolus of PBS was injected over 1 second. From the resulting pressure increase and decay, the compliance and outflow facility were obtained using (1) and (4) respectively. Three injections were performed, and the measurements were compared with those taken using an independent perfusion system which used a gravity-feed technique [28].

#### Enucleated porcine eye closed-loop IOP control

Enucleated porcine eyes obtained from a local abattoir were used to demonstrate closed-loop control of IOP, in which the pump flow rate was automatically adjusted to bring IOP to a desired setpoint. Porcine eyes were chosen because they have a size, compliance, outflow facility, and other characteristics similar to those of human eyes. The eye was prepared by removing the extraocular fat using surgical scissors. The eye was submerged in PBS to the height of the corneal limbus, with the cornea facing up, and this free surface was used as the datum for the pressure transducer. The eye was connected to the syringe pump and pressure transducer by inserting two 30-gauge needles into the anterior chamber, as shown in Fig 1. The cornea was covered by a thin piece of PBS-soaked gauze to maintain corneal hydration.

IOP was raised in 1 mmHg steps (5 sec per step), then lowered in 5 mmHg steps (10 sec per step). The syringe pump flow rate was controlled via proportional feedback control, with gain *K*_p_ = 500 μL min^-1^ mmHg^-1^. For each step, the settling time was calculated as the time after which IOP remained within ±10% of the step size from the set point.

### *In vivo* tree shrew study

This study was carried out in strict accordance with the recommendations made in the NIH Guide for the Care and Use of Laboratory Animals. All procedures were approved by the University of Alabama at Birmingham (UAB) Institutional Animal Care and Use Committee (approved APN: 140910226).

Four adult (at least 6 months old) tree shrews (*Tupaia belangeri*) were obtained from the tree shrew colony maintained at UAB. Ocular compliance and outflow facility were measured in two animals, video of the retinal vasculature was recorded while manipulating IOP in one animal, and spontaneous ICP was recorded in one animal. All experiments were performed under anesthesia, which was induced using a ketamine/xylazine cocktail (100 mg/kg ketamine, 7.5 mg/kg xylazine, intramuscular) and maintained via continuous inhalation of 1% isoflurane.

#### Anterior chamber cannulation

Prior to cannulation of the anterior chamber, the eyelid was secured open using a 9–0 Prolene suture. The cornea was moistened via continuous drip of sterile PBS. The pupil was dilated by applying one drop of 10% phenylephrine to the cornea. Two beveled 30-gauge needles were inserted into the anterior chamber near the lateral corneal limbus using a three-axis micromanipulator arm, to an insertion depth of approximately 1 mm. The needles were held parallel with 1 mm pitch using an aluminum V-groove fixture, with needle bevel openings facing away from one another. The micromanipulator arm held both needles in place throughout the procedure.

#### Tree shrew ocular compliance and outflow facility measurement

To measure ocular compliance and outflow facility, a 3 μL bolus of PBS was injected into the anterior chamber over 1 second. From the resulting IOP increase and decay, the ocular compliance and outflow facility were calculated using Eqs 1 and 4 respectively. Three injections were performed in the left eye of each of two animals.

#### Retinal venous pulsation monitoring

As a proof of concept, the system was used to manipulate IOP to demonstrate that changing IOP can elicit visible changes in the caliber of the retinal veins. IOP was gradually decreased from 10 mmHg to 3 mmHg over approximately 70 seconds by manually lowering a PBS-filled hydrostatic pressure reservoir connected to the eye. The optic nerve head was observed using a slit lamp (Zeiss SL 130) modified with a camera to record video (framerate 30 Hz), with a 78 diopter lens manually positioned in front of the eye.

**Fig 3:**
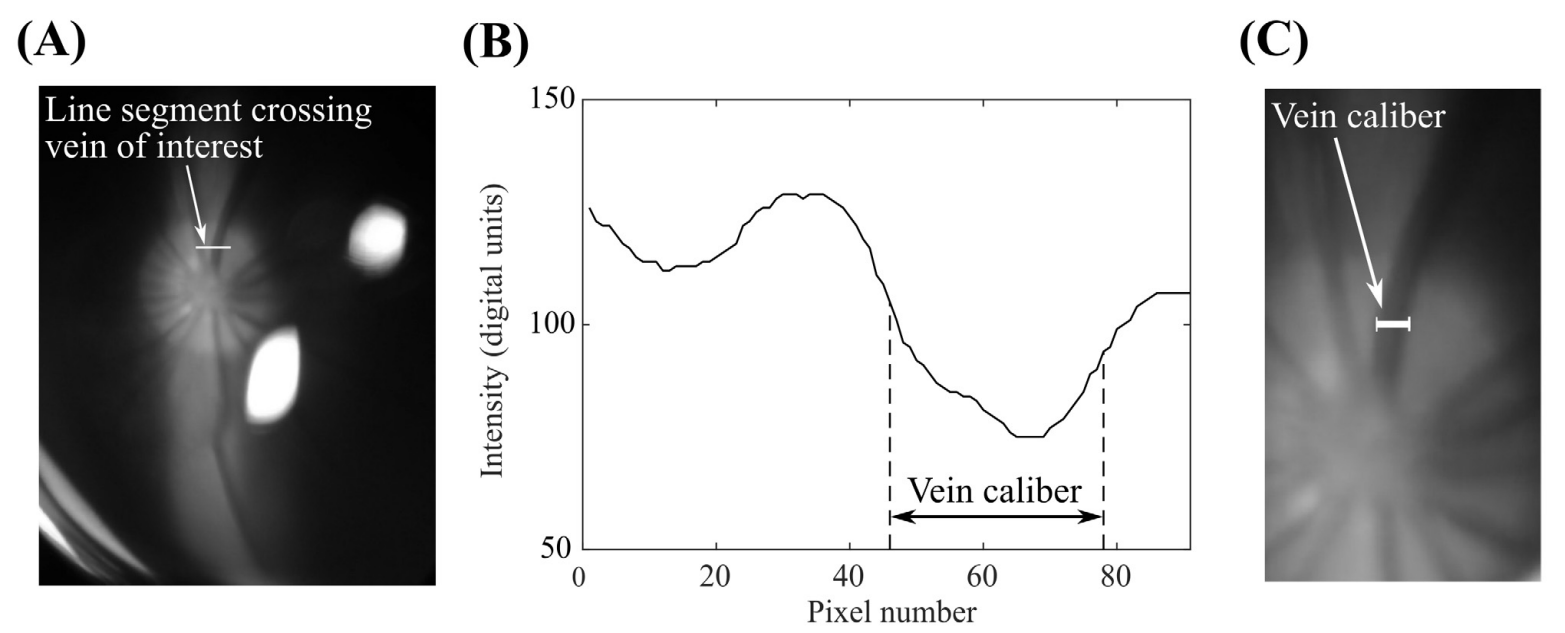
Approach used to automatically measure the caliber of a selected retinal vein. (a) View of the tree shrew optic nerve head, with a line segment crossing the selected vein. (b) Green channel intensity was plotted for the selected pixels on this line segment; vein caliber was taken as the full-width-at-half-maximum of the intensity plot. (c) The resulting vein caliber measurement (*overlaid*) corresponded well to the expected vein caliber.

Video of the optic nerve head was analyzed to quantify changes in the caliber of a selected retinal vein (Fig 3). A line segment crossing the vein of interest was manually identified (Fig 3A); the same line segment was used for each frame of the video. The green channel intensity, selected due to the increased contrast between the vein and the optic nerve head in this channel, was plotted for the pixels along the line segment of interest (Fig 3B). The vein caliber was calculated as the full-width-at-half-maximum of the green channel intensity peak corresponding to the vein. This measurement corresponded well to the expected vein caliber (Fig 3C). These calculations were repeated for each frame of the video, then the resulting vein caliber trace was filtered using a moving average filter with a 1 second window and normalized to the maximum measured vein caliber. The baseline heart rate in an anesthetized tree shrew is approximately 180 bpm, so each 1 second window averages the vein caliber through approximately three cardiac cycles.

#### Spontaneous intracranial pressure measurement

Spontaneous intracranial pressure was measured in a tree shrew not used for IOP manipulation experiments. After induction of anesthesia, the fur overlying the top of the skull was removed using hair clippers, and the animal was secured in a stereotaxic frame. A 2.5 cm midline incision was made through the skin to expose the midline structure and bregma. Cotton tipped applicators soaked in PBS were used to reflect the skin laterally and clean the connective tissue from the skull. A Dremel drill was used to create a hole in the skull, and a 23-gauge cannula attached to a pressure transducer (Honeywell 142PC01G) was lowered into the lateral ventricle. ICP readings were obtained by connecting the pressure transducer to a PowerLab 8/35 data acquisition system (AD Instruments) running LabChart 7.0 software. Appropriate placement of the cannula was determined by lowering the cannula while monitoring the ICP waveform: once the cannula tip passed through the cortex and entered into the lateral ventricle, a clear respiratory waveform could be observed. Once a consistent respiratory and cardiac waveform was obtained, cyanoacrylate glue was used to secure the cannula and create a watertight seal. To obtain a second independent recording of ICP from another location, another 23 gauge cannula attached to a pressure transducer was inserted into the subdural cavity in a similar manner and also secured with cyanoacrylate glue.

## Results and Discussion

### Instrument characterization

#### System volumetric compliance

The volumetric compliance of the system was measured as 0.035 ± 0.002 μL/mmHg (mean ± SD, *n* = 4). This is less than the compliance of a typical human eye by a factor of roughly thirty [29], providing an acceptable signal-to-noise ratio for clinical ocular compliance measurements. Additionally, it is less than our tree shrew ocular compliance measurements by a factor of ten, again providing an acceptable signal-to-noise ratio.

#### Artificial eye measurements

Using our system, the compliance of the artificial eye was measured as 0.237 ± 0.002 μL/mmHg (*n* = 3; see also S1 Dataset). This compliance measurement compares favorably with measurement of 0.227 ± 0.014 μL/mmHg (*n* = 4) taken using the independent perfusion system.

Using our system, the outflow facility of the artificial eye was measured as 21.2 ± 1.3 nL min^-1^ mmHg^-1^ (*n* = 3), and the independent perfusion system obtained an outflow facility measurement of 17.0 ± 0.3 nL min^-1^ mmHg^-1^ (*n* = 4). The average measurements are comparable, but somewhat larger variability was observed between measurements than was observed in the independent perfusion system.

To check the assumption of negligible fluid outflow from the artificial eye during the 1 second injection, outflow volume was estimated from the measured outflow facility (21.2 nL min^-1^ mmHg^-1^) and the maximum pressure that occurred during the bolus injections (14.7 mmHg), resulting in an estimated of 5 nL of outflow during the injection, or 0.5% of the total injected volume. This outflow volume was considered negligible.

#### Enucleated porcine eye closed-loop IOP control

**Fig 4:**
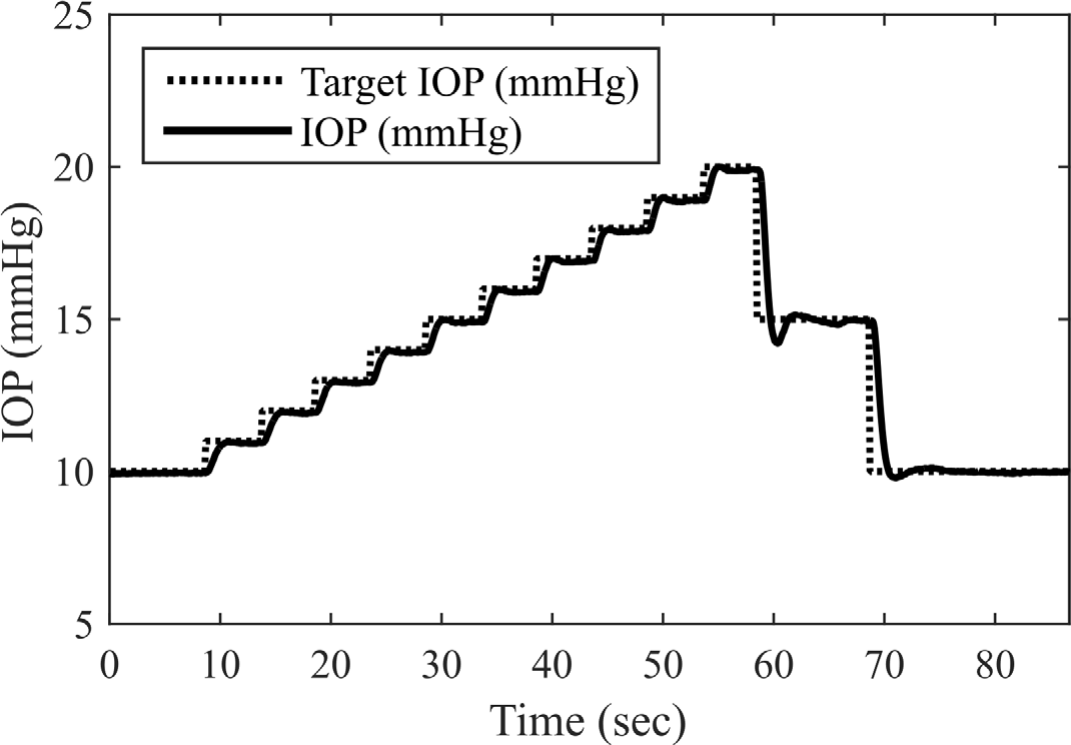
The system enabled closed-loop control of IOP, demonstrated here in an enucleated porcine eye.

Settling times following step changes in setpoint IOP were rapid (1.23 ± 0.35 sec), with steady-state errors < 0.10 mmHg (Fig 4; see also S2 Dataset). Although performance could likely be improved via further tuning of the controller gains, this dynamic response was judged to be suitable for a prototype instrument.

### *In vivo* tree shrew study

#### Ocular compliance and outflow facility measurement

**Fig 5:**
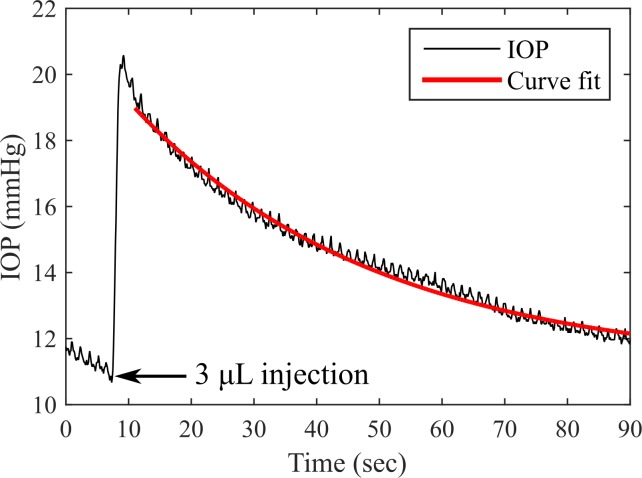
Bolus injections were used to measure ocular compliance and outflow facility in tree shrews.

Ocular compliance and outflow facility were measured in the eyes of living tree shrews by injecting 3 μL of PBS into the anterior chamber over 1 second and recording the resulting IOP increase and subsequent decay (Fig 5; see also S3 Dataset). Superimposed on the IOP increase and decay are visible physiological pressure oscillations resulting from respiration and the ocular pulse, characteristic of *in vivo* IOP measurements.

Ocular compliance was measured as 0.312 ± 0.019 μL/mmHg in the first animal and 0.231 ± 0.056 μL/mmHg in the second (*n* = 3 injections per animal; see also S4 Dataset). While tree shrew ocular compliance measurements have not previously been reported to our knowledge, these measurements are reasonable in that they follow the general trend that larger eyes are more compliant, lying between values reported in mice (0.086 ± 0.017 μL/mmHg, [30]) and rabbits (3.03 ± 0.65 μL/mmHg, [31–33]).

Outflow facility was measured as 0.085 ± 0.033 μL min^-1^ mmHg^-1^ in the first animal and 0.303 ± 0.098 μL min^-1^ mmHg^-1^ in the second (*n* = 3 injections per animal; see also S4 Dataset). Again, these measurements were reasonable in light of values reported in mice (0.0125 ± 0.0037 μL min^-1^ mmHg^-1^, [30, 34]) and rabbits (0.257 ± 0.055 μL min^-1^ mmHg^-1^, [35–36])

To check the validity of the assumption of negligible aqueous outflow during the 1 second injection, the outflow volume during the injection was estimated from the greater outflow facility measurement (0.303 μL min^-1^ mmHg^-1^) and the maximum IOP observed during the bolus injections (24.5 mmHg, Fig 5), resulting in an estimate of 120 nL of outflow during the injection, or 4% of the injected volume. This was considered negligible, but indicates that these measurements slightly overestimate the true ocular compliance.

#### Retinal venous pulsation monitoring

**Fig 6:**
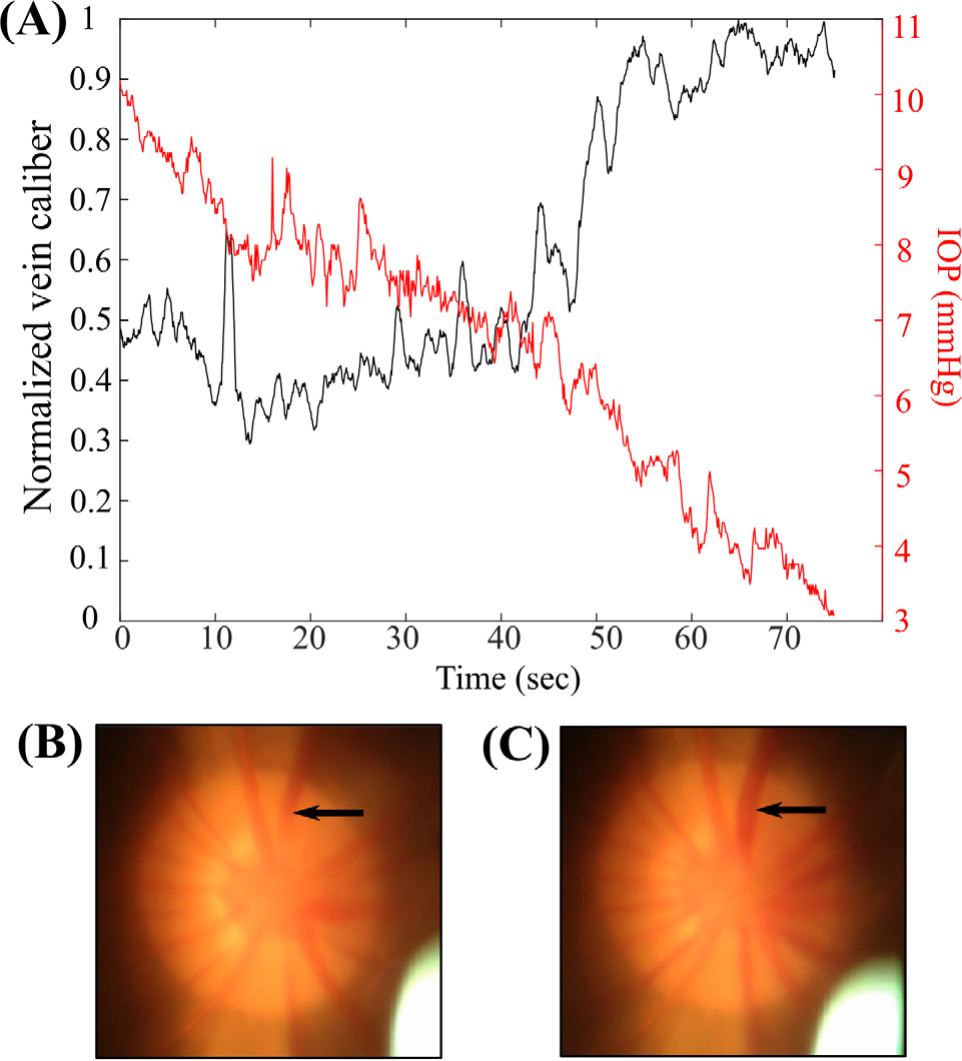
Retinal vein caliber increased sharply as IOP was lowered past a threshold value. (a) Retinal vein caliber was approximately constant while IOP was above 7 mmHg, increase sharply as IOP was decreased from 7 mmHg to 5 mmHg, then was approximately constant as IOP was decreased further to 3 mmHg. (b) Retinal vein in its baseline state. (c) Retinal vein in its dilated state.

As IOP was decreased from 10 to 3 mmHg over approximately 70 seconds, retinal vein caliber remained approximately constant while IOP was above 7 mmHg, increased sharply as IOP was decreased from 7 mmHg to 5 mmHg, then was approximately constant as IOP further decreased from 5 mmHg to 3 mmHg (Fig 6; see also S1 Video and S5 Dataset). The indicated vein visibly pulsated as it became dilated. Some of the noise in the vein caliber trace is likely due to relative motion between the camera and the tree shrew, causing the position of the vein within the field of view to change. Of note, the mechanism of human retinal venous pulsation proposed by Levine [14] does not appear to directly apply in the tree shrew, since rapid dilation of the retinal veins was observed as IOP was lowered, rather than loss of pulsations.

#### Spontaneous intracranial pressure measurement

**Fig 7:**
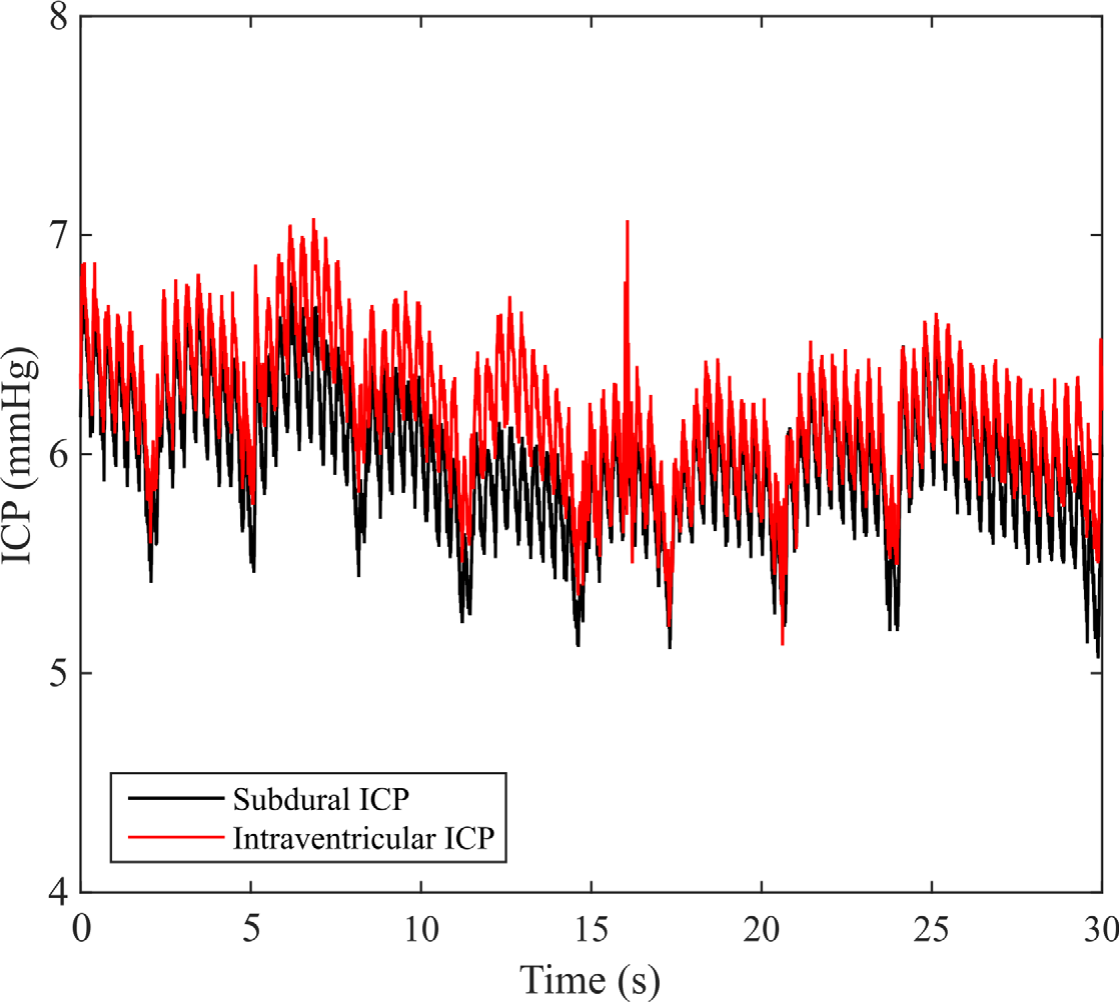
Subdural and intraventricular recordings of tree shrew spontaneous ICP oscillated between 6–7 mmHg.

Intraventricular and subdural measurements of spontaneous ICP oscillated between 6–7 mmHg (Fig 7; see also S6 Dataset). As expected, cardiac and respiratory pressure oscillations were visible in both the intraventricular and subdural ICP signals.

## Conclusions

The system as implemented shows excellent accuracy and dynamic response, both essential qualities required for eventual clinical implementation. The approach that we have developed could be adapted to a small, hand-held device suitable for use in a clinical setting. Spontaneous retinal venous pulsation is present in 94% of patients [13], suggesting that with further development, the retinal venous pulsation method could conceivably replace highly-invasive ICP measurement procedures in many patients. Perhaps more importantly, this technique estimates ICP at the level of the optic nerve, i.e., the post-laminar pressure, which is the relevant pressure in glaucoma management. Because lumbar puncture measures CSF pressure a significant distance from the optic nerve, these measurements are subject to a variety of anatomical and fluid level influences that can vary significantly between patients, largely eliminating the utility of a non-ophthalmic approach.

The work of Golzan et al. [17] on ICP estimation using retinal venous pulsation suggests that with improved methods of IOP manipulation, the retinal venous system could provide very accurate estimates of ICP. Our system meets this need by providing accurate and precise IOP manipulation. Further, we demonstrate that this system provides visible changes in the retinal vasculature, which suggest its utility for studying retinal venous pulsation in vivo. Open questions remain. For example, since changes in venous pulsation appear to occur over a range of IOP values, albeit a very narrow one, it is as yet unknown which part of this IOP range corresponds to ICP. While theoretical models of retinal venous pulsation exist [14], to date these models have only considered steady-state behavior, and thus do not provide insights into the onset or cessation of pulsation. Rather than relying on a theoretical model, we envision performing a “calibration” in which ICP is directly measured during IOP manipulations, to identify which portion of the IOP waveform corresponds to ICP. While this is a natural extension of this research, it is beyond the scope of the present work, which has focused primarily on technology and methods development.

In addition to the possibility of measuring ICP, the system has the capability of determining ocular compliance and outflow facility, which are of interest in basic glaucoma research and clinical management. It has become increasingly obvious that biomechanical factors are responsible for the retinal ganglion cell damage characteristic of glaucoma [37–38], and thus the ability to measure biomechanical properties of the eye *in vivo* is an important goal to improve management of patients with this common disease. While the clinical importance of measuring outflow facility in glaucoma patients has been recognized for decades [39–40], the standard measurement approach, tonography, dates to the mid-twentieth century and is noted for its relatively poor accuracy [41] and how difficult it is to perform well. Thus, although accurate measurements of outflow facility would be invaluable in diagnosing and managing glaucoma, this technology is generally unavailable and has fallen out of clinical use worldwide.

These advantages come at the cost of some level of invasiveness, since our IOP manipulation approach requires penetration of a small needle through the peripheral cornea. However, procedures involving access of the anterior or posterior compartment of the eye are used routinely in patient care, and cannulation of the anterior with a small needle offers minimal risk to vision or to the eye. Although perhaps not appreciated by the general public, intraocular injections are one of the most commonly performed ocular procedures [42], particularly for injection of anti-vascular endothelial growth factor medications for the treatment of age-related macular degeneration [43]. The cornea has a remarkable ability to re-seal after puncture, a property that is already routinely used clinically, e.g., in paracentesis, in which the cornea may be punctured using needles as large as 26G [44–45]. In a clinical implementation of the system, it would be desirable to use a single, smaller needle (e.g., 33G) to simultaneously measure IOP while injecting or withdrawing fluid. The use of a single needle could be achieved by calibrating for the needle resistance and system compliance, in order to relate the measured pressure to IOP. Given these factors, the procedure we describe would carry lower risks than current invasive options for ICP measurement.

We recognize that the techniques and experiments reported herein are preliminary, and will require much additional development and testing before clinical deployment. However, we feel that the results are sufficiently encouraging that further development is warranted.

## Supporting Information

S1 Dataset Artificial eye ocular compliance and outflow facility measurements from our system and the gravity-feed system. (XLSX)

S2 Dataset Closed-loop control of IOP in an enucleated porcine eye. (XLSX)

S3 Dataset Tree shrew IOP vs. time traces, used for ocular compliance and outflow facility calculations. (XLSX)

S4 Dataset Summary of tree shrew ocular compliance and outflow facility calculations. (XLSX)

S5 Dataset IOP and retinal vein caliber traces. (XLSX)

S6 Dataset Subdural and ventricular recordings of tree shrew spontaneous ICP. (XLSX)

S1 Video Video of tree shrew retinal vein dilation, from which vein caliber was measured (Fig 6). (MP4)
